# Digital health tools to support parents with parent-infant sleep and mental well-being

**DOI:** 10.1038/s41746-022-00732-4

**Published:** 2022-12-21

**Authors:** Helen L. Ball, Alice-Amber Keegan

**Affiliations:** Durham Infancy & Sleep Centre, Department of Anthropology, Durham University, Durham, UK

**Keywords:** Biological anthropology, Risk factors

## Abstract

Digital technology is increasingly important in people’s lives, particularly for new parents as it allows them to access information, stay connected to peers and offers them seductive solutions for improving infant sleep and parental well-being. Digital technology has been developed to support parents in the following four ways: (1) providing digital information on infant sleep, (2) offering targeted support for night-time care, (3) managing infant sleep and (4) monitoring infant sleep and safety. Evidence on the effectiveness of these strategies is varied and there are concerns regarding the reliability of information, use of personal data, commercial exploitation of parents, and the effects of replacing caregiver presence with digital technology.

## Introduction

Millennial parents (individuals born between 1981 and 1996^1^) who have grown up alongside the digital age and who are now becoming parents are increasingly and consciously using digital tools to support night-time parenting. Offering new and seductive solutions to the longstanding stressors of new parenthood such as sleep deprivation, work-life balance, and meeting familial/social expectations, digital parenting tools are now ubiquitous. Given their potential to influence attitudes and practices about normal and safe infant sleep, it is vital that the application of digital tools to night-time infant care is carefully appraised. In this commentary we critically evaluate the types of digital health tools available to new parents and offer an anthropologically informed view of the positive and negative aspects of their use.

## Human sleep in a digital world

Sleep patterns in Western industrialised societies have shifted considerably over recent centuries^2^. Nowadays, children and adults exhibit a monophasic sleep pattern of one long consolidated sleep period at night, and normative expectations of 8-h uninterrupted sleep prevail^3,4^. Social changes over the past century such as urbanisation, widespread artificial lighting, introduction of shift-work, and the availability of screen-based digital media have all contributed to a changing sleep landscape^4,5^. Rapid development of digital technology in the twenty-first century has produced what Coveney^6^ describes as a ‘wired awake’ world, and with the popularity of phone applications and wearable devices sleep has become a heavily scrutinised aspect of modern life^6–8^.

Normative sleep duration for infants is hugely variable, with some new-borns sleeping 22 h per day and others 8 h^9^. Born without a day/night rhythm the infant circadian clock develops throughout the first year, so night-time sleep is not indicative of total infant sleep patterning or duration. Furthermore, the evolved biology of human infants requires close contact from a caregiver to support the establishment of breastfeeding, infant physiological regulation, and the development of the parent-infant relationship^10^. Sleep in early infancy is primarily biologically driven, and conflicts with adult sleeping practices that are culturally shaped.

When juxtaposed with contemporary adult sleep expectations, the sleep disruption experienced with a new baby becomes one of the most difficult aspects of parenting, and one for which first-time parents are often ill-prepared^11^. Fragmented extended families reduce opportunities for emotional and practical support, leaving new parents feeling overwhelmed, and even triggering depression and the breakdown of relationships^12^. Digital tools focussed on infant sleep are marketed to new and prospective parents as offering opportunities to reduce the stress of infant caregiving and improve parental well-being.

## How does digital technology interact with parental and infant sleep?

Despite existing in diverse forms—from informational websites, blogs and podcasts, social media forums, apps and digital trackers, to monitors, AI-enabled responders, and internet-connected cribs—digital tools focussing on sleep primarily fall into four domains: (a) to help parents better understand their infant’s sleep, (b) to support parents in providing night-time care, (c) to assist parents in ‘managing’ their baby’s sleep and (d) to facilitate remote monitoring of infant sleep. Below we explain how these domains are distinguished and map them against the WHO classification of digital interventions.

A. **Providing digital information on infant sleep**
  This group of tools corresponds to the WHO classification 1.2.1: digital interventions that transmit untargeted health information to an undefined population and may take the form of websites, blogs, apps, and social media groups that offer rapid access to information and the experiences of others 24 h a day. Parents find such online sources of information on infant sleep attractive because they are ‘always available’, ‘up-to-date’ and ‘fast’^13^. Website analytics show that Durham University’s online information tool Basis (Baby Sleep Information Source), for instance, is accessed frequently at night from mobile devices as parents seek to understand their baby’s night-waking or insistence on sleeping like a limpet attached to a parent’s body^14^. Although there are a number of publicly available, evidence-informed websites that provide information on infant sleep to parents in different geographic locations (e.g., BabySleep.com, BasisOnline.org.uk, LittleSparklers.org), parents may find them difficult to discern from, other, less reliable sources such as ‘mommy blogger’ websites and YouTube ‘infomercials’ for popular parenting products. These are often thinly disguised money-making ventures linked to sponsorships and book deals^15^ and some of these commercially motivated online sources offer misinformation (e.g., unsafe sleep arrangements) that parents lacking sophisticated appraisal skills may not detect^16^.
  Many millennial parents are consumers of parenting content shared on social media by peers and influencers. Social media platforms encourage the creation of aesthetically pleasing sleep environments, which focus on comfort, cosiness, and beauty rather than practicality or safety. An analysis of 1563 Instagram images found that only 7% were consistent with American Academy of Paediatrics (AAP) safe sleep guidelines^17^, with the presence of hazardous bedding, and sleeping in a non-recommended position the most common concerns. Such images reinforce norms of unsafe sleeping practices and create aspirational infant sleep environments sponsored by infant product manufacturers, marketing products that are financially inaccessible to many.
  Yet social media and virtual online spaces that aim to transmit information also facilitate the creation of supportive communities with others who share the same schedules, lifestyles and parenting values. The prevalence of online infant feeding support groups allows mothers to find refuge in online spaces outside of the temporal constraints of their wider physical and social worlds. Many mothers with young infants use social media, such as Facebook groups, to meet others living nearby and alleviate feelings of isolation^18^, supporting maternal well-being.
B. **Offering targeted support for night-time care**
  This second group of tools correspond to the WHO classification 1.1.2: digital interventions that transmit targeted health information to client(s) based on health status or demographics. The use of e-learning platforms^19^ and phone apps to deliver infant sleep safety messaging^20^ or provide guidance about infant sleep development are examples of digital tools/interventions delivering specific information to targeted recipients. Web-based and app-based infant sleep tools and interventions have multiplied exponentially over the last decade and, like untargeted information sources, many digital support tools are commercially motivated^21^. Sometimes these platforms are sponsored by a manufacturer whose products are recommended as part of the support intervention^22^, or are funded via the use of adverts or in-app purchases.
  Digital tools such as phone apps are expensive to design, develop, and keep up-dated, and few app-providers can afford to undertake this work without it generating income. Traditionally commercial apps are funded through subscription access model, where a basic support app can be accessed at no cost to the user in the hope they will choose to pay for premium ‘unlockable’ content after their free taster. Commercial apps can pose privacy risks by selling personal data to third parties or providing content influenced by commercial gain^23^. It is reported that online marketers pay more for pregnant women’s browsing data than other internet users due to their increased spending activity^24^. Formula companies have been shown to use parenting apps to target advertising to parents, breaching the WHO International Code of Marketing of Breast Milk Substitutes^25^. Likewise, some companies offering baby boxes in North America and UK created ‘educational’ websites offering safe sleep information that promoted various products to families who signed up for a baby box^26^.
  In contrast to their commercially supported counterparts, evidence-based apps made freely available by academic and charitable organisations, tend to lack user engagement and user-friendly interfaces due to the prohibitive costs of design and development, leading many parents to favour commercial versions offering greater interactivity and customisation^23,27,28^. One way that some researchers have funded targeted mHealth interventions is through collaboration with commercial organisations. Industry funded randomised trials have demonstrated that customisable internet-based and smartphone applications, created by researchers and funded by commercial support were effective in improving parent-perceived infant sleep problems^21,22^. The success of free, evidenced-based parenting apps like Baby Buddy, provided by the UK-based charity Best Beginnings, demonstrate that with the appropriate support and funding from philanthropic organisations and health-care providers, targeted evidence-based apps can be as well-designed as commercial apps and provide targeted parenting support^29,30^ without the associated ethical concerns.
C. **‘Managing’ infant sleep**
  This group of digital interventions do not appear in the WHO classification, but are digital upgrades to analogue products used by parents in the hope of reducing infant sleep fragmentation and enhancing duration. These devices range from noise machines to responsive bassinets featuring artificial intelligence (AI), with many claiming to settle infants without the need for caregiver presence.
  Devices producing white noise, often marketed as ‘sound sleep aids’ or infant ‘sleep machines’^31^, promise to soothe and settle infants to sleep using ambient or white noise. Many are embedded within cuddly toys, enhanced with cry sensors that respond by playing a soothing tune. There is some evidence that white noise is effective in inducing sleep in young infants^32^, however, there are concerns that sound levels in infant sleep machines may be damaging to infant hearing and auditory development^31^. Regardless of the impact on infant sleep, these devices may disturb parental sleep when infants room-share, prompting parents to prematurely move infants into a separate room, as well as normalising the use of soft toys in infant sleep environments contrary to safe sleep guidance.
  Cry sensors and sound machines are also embedded within ‘smart’ or AI-enabled bassinets. Marketed as ‘virtual babysitters’, these devices respond to infant waking by rocking, vibrating, and playing soothing sounds^33^. A growing number of these devices include built in video and audio monitors linked to phone apps that display data on infant sleep continuity and duration as well as sending push notifications to parents when their baby wakes. Questions around the effects of these devices on breastfeeding outcomes, and the consequences for infant social development of using AI-technology to reduce parental touch, soothing and other aspects of responsive parental care have not been addressed.
D. **Monitoring infant sleep and safety**
  This final group of tools corresponds to the WHO classification 1.4.2: digital interventions for self-monitoring of health or diagnostic data by client. Smart technology that allows parents to remotely monitor their infant’s physiology has become hugely popular in recent years, promising reassurance to parents and decreasing ‘negative touch’—touch that serves to reassure parents with no discernible benefit to the infant^34^. As well as delivering detailed sleep tracking information these products provide up-to-the minute data on infant physiological state, sending alerts of physiological irregularities. These devices claim to serve multiple purposes; to support infant sleep safety, to relieve parental anxiety and to address caregiver-identified infant sleep problems. It is claimed this technologically enhanced reassurance improves the quality of parental sleep and reduces “unnecessary” waking to check on an infant. Manufacturer funded research has, for instance, found that parents self-report better quality sleep for themselves when using a smart sock device^35^, however no objective studies have tried to confirm this.
  While these products are marketed to parents as opportunities to buy reassurance, some reports suggest they can paradoxically increase parental anxiety due to compulsive observation of their infant’s physiological state^34^. Another risk of digital monitoring tools is that they foster a false sense of complacency when parents believe the monitor will compensate for their lack of vigilance or an unsafe sleep environment. Little is known about whether these devices enable intervention should the infant experience a physiological crisis, and all these devices carry disclaimers that they cannot prevent SIDS^36^. Although widely available and used by parents, there are concerns about the suitability of these products for use by the public; one digital infant monitoring product was removed from the US market when the Food and Drugs Administration (FDA) warned that the product was a medical device requiring FDA approval to be marketed and sold^37^.
  A common problem with reliance on smart devices is the normalisation of parent-infant separation; the existence of digital monitors, cribs and toys implies that infant caregiving can be outsourced to technology, allowing parents to rapidly return to their pre-baby lives. Promotion of these products does not consider the importance of the evolved biological relationship between mother and baby, or the dyadic physiological feedback loops, which serve to support the initiation and establishment of breastfeeding and consolidate parent-infant bonding^38–40^. These devices reinforce cultural narratives around good and bad types of parent-infant sleep and touch, and empower parents to leave their infants unattended.

## Conclusion

There is value in the use of digital tools for infant sleep information provision, but we recognise that digital tools are only as good as the information that is fed into them; not all parents have the skills to discern legitimate information or websites from clickbait or advertising. Social media and infant sleep apps can be hugely influential in shaping cultural narratives around night-time infant care, but many are driven by commercial motives rather than the health and well-being of parents and infants. We see value in digital tools for providing parent support such as no or low-cost online communities, and advert-free, evidence-informed digitally delivered support interventions. The more expensive digital devices have the potential to undermine responsive parenting, encourage night-time separation of parents and babies, and exploit parental anxieties. To be beneficial, new digital tools should enhance infant care skills, such as educating parents about infant cues and encourage parents to physically interact with their baby. We encourage parents to use their phones, tablets and other digital devices as information sources to learn about their babies’ sleep needs and their sleep development, and to connect with other parents to exchange knowledge and experiences, rather than as tools to pacify their baby remotely, and substitute for parental presence.

### Reporting Summary

Further information on research design is available in the Nature Research Reporting Summary linked to this article.

## Supplementary information

Reporting Summary
